# The Hard X-ray Nanoprobe beamline at Diamond Light Source

**DOI:** 10.1107/S1600577521002502

**Published:** 2021-04-09

**Authors:** Paul D. Quinn, Lucia Alianelli, Miguel Gomez-Gonzalez, David Mahoney, Fernando Cacho-Nerin, Andrew Peach, Julia E. Parker

**Affiliations:** a Diamond Light Source, Harwell Science and Innovation Campus, Didcot, Oxfordshire OX11 0DE, United Kingdom

**Keywords:** Hard X-ray Nanoprobe, multimodal techniques, nano-XRD, nano-XRF, nano-XANES, ptychography, spectro-microscopy

## Abstract

The Hard X-ray Nanoprobe beamline, I14, at Diamond Light Source is a new facility for nanoscale microscopy. The beamline was designed with an emphasis on multi-modal analysis, providing elemental mapping, speciation mapping by XANES, structural phase mapping using nano-XRD and imaging through differential phase contrast and ptychography. The 185 m-long beamline operates over a 5 keV to 23 keV energy range providing a ≤50 nm beam size for routine user experiments and a flexible scanning system allowing fast acquisition. The beamline achieves robust and stable operation by imaging the source in the vertical direction and implementing horizontally deflecting primary optics and an overfilled secondary source in the horizontal direction. This paper describes the design considerations, optical layout, aspects of the hardware engineering and scanning system in operation as well as some examples illustrating the beamline performance.

## Abstract 2

The design and operational performance of beamline I14, the Hard X-ray Nanoprobe at Diamond Light Source, is presented.

## 1. Introduction   

Probing heterogeneous complex systems with a focused X-ray beam to measure local variations in composition, structure and morphology has been shown to be a powerful tool in diverse scientific areas including life science (Hémonnot & Köster, 2017 ▸), cultural heritage (Cotte *et al.*, 2018 ▸) and materials science (Johannes *et al.*, 2017 ▸; Yu *et al.*, 2018 ▸).

Recent years have seen rapid advances in the field of soft and hard X-ray microscopy, driven by the emergence of ultra-brilliant synchrotron sources (Tavares *et al.*, 2014 ▸; Liu *et al.*, 2014 ▸), advances in instrumentation (Holler *et al.*, 2018 ▸; Schroer *et al.*, 2017 ▸; Deng *et al.*, 2019 ▸; Nazaretski *et al.*, 2014 ▸) and new imaging methodologies (Deng *et al.*, 2018 ▸; Yu *et al.*, 2018 ▸). Hard X-rays in particular have benefited from significant advances in hard X-ray focusing optics using either reflective (Yamauchi *et al.*, 2011 ▸), refractive (Seiboth *et al.*, 2017 ▸; Patommel *et al.*, 2017 ▸), diffractive (Huang *et al.*, 2013 ▸; Suzuki *et al.*, 2010 ▸; Mimura *et al.*, 2010 ▸) and waveguide (Krüger *et al.*, 2012 ▸) schemes to achieve beam sizes from 7 nm to 50 nm.

These advances, coupled with the scientific demand for higher spatial resolution, have led to the construction of a number of hard X-ray nanoprobes at synchrotrons worldwide providing a broad range of techniques and beam-sizes (Chang *et al.*, 2013 ▸; Suzuki *et al.*, 2013 ▸; Nazaretski *et al.*, 2017 ▸; Winarski *et al.*, 2012 ▸; Martínez-Criado *et al.*, 2016 ▸; Chen *et al.*, 2014 ▸; Somogyi *et al.*, 2015 ▸; Schroer *et al.*, 2010 ▸; Johansson *et al.*, 2013 ▸; Tolentino *et al.*, 2017 ▸).

The science drivers for each beamline play a large part in the choice of focusing optic and optical layout, which in turn influences the working distance, beam size, energy range and chromaticity. Broadly speaking, multilayer Laue lenses or multilayer Kirkpatrick–Baez mirrors (KBs) can offer the highest spatial resolutions [currently < 10–20 nm (Huang *et al.*, 2013 ▸)] but are primarily fixed-energy optics. Hard X-ray zone plates can typically offer resolutions down to 10 nm (Mohacsi *et al.*, 2017 ▸) with a broader energy range, but with a need to adjust for energy-dependent focal lengths and generally reduced performance at higher energies. Reflective KB mirrors are achromatic, which makes them the most flexible and most suitable to spectroscopic studies and broad-energy operation; however, with a 40–50 nm beam size they offer comparatively lower spatial resolution. The science case for the Hard X-ray Nanoprobe, I14, at Diamond Light Source (DLS) is centred on spectro-microscopy and *in situ* operation in a broad range of areas including energy and battery materials, zircalloy cladding from the nuclear industry or radionuclides in environmental cells. In order to meet this demand, the nanoprobe uses a reflective KB system which operates over a 5 keV to 23 keV energy range and yields a nominal ≤50 nm beam (at 12 keV) for multi-modal analysis. The beamline provides spatial mapping of elemental composition by X-ray fluorescence (XRF), speciation by X-ray absorption near-edge spectroscopy (XANES spectro-microscopy), structural phase by nano-X-ray diffraction (nano-XRD), and phase or electron density through differential phase contrast (DPC) and ptychography, but with a particular emphasis on spectro-microscopy. In this paper, we report on the overall design, operational characteristics and key examples of operational performance.

## 2. Beamline optical design   

The beamline design must sufficiently de-magnify the source while accounting for achievable levels of mechanical stability, allow enough working distance to meet the experimental requirements, provide a coherent beam for diffraction-limited focusing and coherent imaging applications, and be robust to energy changes during operation.

Nano- and micro-focusing optical arrangements can be single stage, in which the source is focused by the nano-focusing optic directly; two-stage, in which the source is focused to an intermediate aperture, acting as a source for the focusing optic; or a combination of the two schemes. The single-stage schemes offer minimal loss of coherence due to imperfect beamline optics, whereas two-stage schemes offer greater control over flux, coherence length and focused spot size (De Jonge *et al.*, 2014 ▸). In practice, the source size at third-generation synchrotrons makes the two-stage solution unavoidable in the horizontal direction but allows some choice in the vertical direction, albeit with an impact on the overall beamline length.

**Figure 1:**
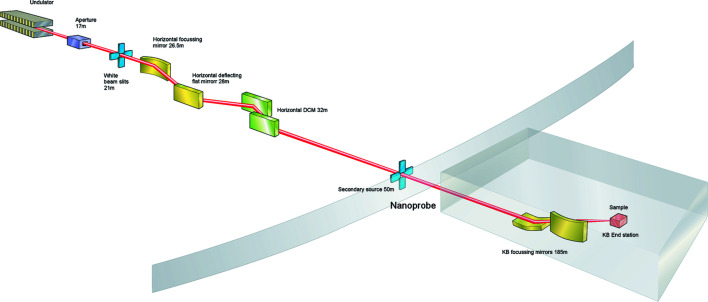
Outline schematic of the Nanoprobe beamline at DLS.

The Nanoprobe at DLS is based on an astigmatic scheme using a combination of single and two-stage focusing. The beamline source is a U23 *in vacuum* undulator. The optics design considered the heatload from a 17.6 mm-period cryogenic permanent magnet undulator which is currently under construction as part of a development program at DLS. Achieving a high-stability beam with 5% or 10% source size movement, with typical source sizes of σ_v_ = 3 µm and σ_h_ = 125 µm in the vertical and horizontal, respectively, is significantly easier if the movement is in the horizontal direction. The beamline uses all-horizontal optics for beam conditioning prior to nano-focusing, exploiting the improved mechanical stability and the more manageable mechanical tolerances afforded by this geometry (Fig. 1 ▸).

Vertically, the source is unmodified and imaged directly by the vertically focusing KB mirror, which is located 185 m from the source. Horizontally, the beam is directed onto slits 52 m from the source, to form a secondary source aperture which is imaged by the horizontal mirror of the KB nano-focusing optic.

The beam is first conditioned by two mirrors, used to control the direction of the source and collimate the beam horizontally. The first mirror (M1, white-beam mirror) deflects the photon beam horizontally and collimates the beam in the horizontal direction. The collimation reduces the natural horizontal divergence (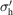) from 25 µrad to approximately 5 µrad. The second mirror (M2, pink-beam mirror) also deflects the beam horizontally but is flat and is used primarily to control harmonic content as well as both the overall beam direction and the fine control. The mirrors have three coating stripes, Si, Rh and Pt, to reduce harmonic contributions across the energy range with both mirrors indirectly water-cooled via a gallium–indium eutectic scheme, employing a gravity fed water-cooling system to minimize vibration.

A horizontal double-crystal monochromator is positioned immediately after the mirrors. The monochromator uses cryogenically cooled Si(111) crystals and can cover a 4.5–25 keV energy range, although the accessible energy range for experiments is reduced to 5–23 keV due the choice of surface coatings and operating angles on the final KB focusing mirrors.

The design of mirror M1 allows us to collimate or focus the source 1:1 onto the secondary source aperture. For the source parameters and optical setup of the Nanoprobe, the differences between these schemes were relatively small. The increased beam size and lower divergence of the collimation scheme produced similar flux at the sample compared with the larger divergence of the focusing scheme. Wavefront propagation and optical simulations of heat bumps and their effect on focusing also indicated that the collimation scheme was more robust. Since collimation also has advantages for monochromatic performance, this scheme was chosen as the default operating mode.

The optics are installed within two hutches inside the main synchrotron building with the first optics hutch containing the mirrors and monochromator and the second optics hutch, located at around 50 m from the source, containing a set of precision slits to define the secondary source. A summary of the optical components and their positions is presented in Table 1 ▸.

### 2.1. Beam duct and external building   

The beam propagates from the synchrotron to the I14 experiment hutch, which is housed in a purpose-built building also housing the complementary Electron Bio-Imaging Centre (eBIC) and Electron Microscopy centre for physical sciences (ePSIC) (Clare *et al.*, 2017 ▸).

The beam duct connecting the synchrotron and external building is constructed of standard concrete with a nominal thickness of 350 mm. Removable concrete lids weighing in excess of 10 tonnes allow access and secured shielding when in place. The beam pipe needs to pass under two sections of road which could not be classed as shielding so additional radiation shielding is provided by a lead ‘cloche’ style arrangement over the pipes under these sections. The duct, when all lids are in place, is completely enclosed with no access so electrical and vacuum equipment were minimized with no pumps installed within the duct, although three sets of double vacuum gauges monitor the vacuum within the beam pipe. This arrangement provides robustness in vacuum monitoring through redundancy while decreasing the need for unplanned access. This vacuum pipe reaches pressures in the region of 3 × 10^−9^ mbar at the middle point, despite the pump stations being located at a distance around 113 m at each end.

**Figure 2:**
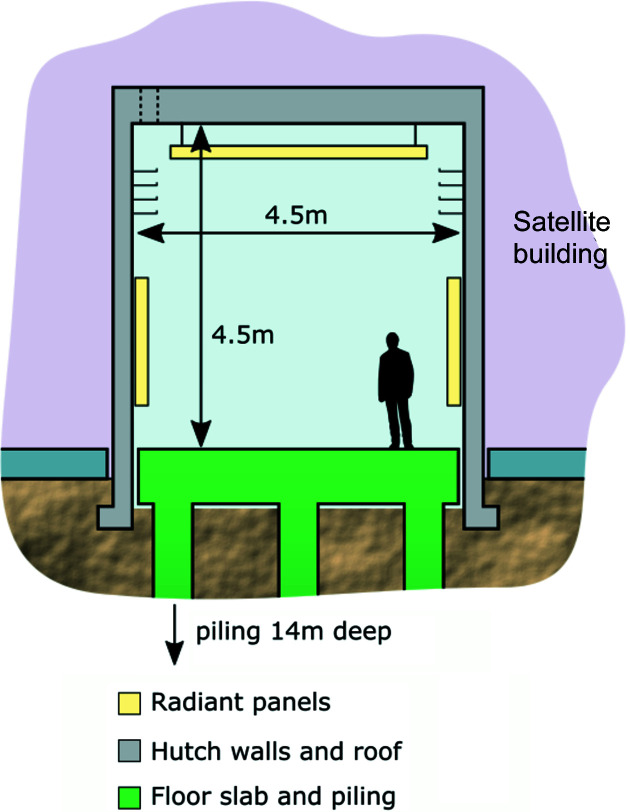
Schematic representation of the main structural features of the hutches in the satellite building. The 800 mm floor slab is supported on piles, buried 14 m deep into the ground with an air gap around the structure. The 300 mm-thick walls are made of reinforced concrete and rest on their own foundations with a 50 mm gap between the hutch floor and the wall, and also between the wall and the building floor.

The structure of the beamline and electron microscope laboratory floors in the external building echoes that of the Diamond storage ring, consisting of an 800 mm-thick slab supported by 14 m-deep piles. This construction design ensures that seasonal changes due to the water table on the chalk ground are similar on both buildings, minimizing the relative displacement between the source and the endstation in the experimental hutch (Fig. 2 ▸).

As depicted in Fig. 2 ▸, the 300 mm-thick walls of the hutch are made of reinforced concrete and rest on their own foundations, which are independent of the piles and the rest of the building. There is a 50 mm gap between the beamline floor and the walls, and between the walls and the building floor. Concrete is very well suited as a construction material for high-stability environments due to its low thermal conductivity and good vibration damping properties. At I14, the thick hutch walls and ceiling provide uniform thermal insulation, structural support for all installations including a 2 T crane and radiation shielding in the photon energy range of the beamline (up to 23 keV).

Excellent thermal management is required to maintain stability. The beamline employs a novel radiant panel cooling system mounted on the ceilings and walls to remove short-term thermal oscillations and limit air pressure fluctuations, while avoiding potential issues with high-volume forced air temperature control. To further aid thermal stability, the experimental hutch is accessed via a vestibule with a self-closing door to minimize the volume of air exchange when opening the hutch door (Cacho-Nerin *et al.*, 2020 ▸).

### 2.2. Beam positioning   

Whilst an arrangement with all-horizontal optics assists with managing vibrations with respect to beam coherence or focusing, it can pose some challenges for long-term stability and operation. In particular, this arrangement results in a coupling of multiple optical elements to the horizontal beam position and, to a lesser extent, the flux. At many beamlines the beam position is used as an indicator of monochromator alignment and is controlled using the monochromator crystal piezo actuators. This scheme would compensate for drift in the pitch of the mirrors before the monochromator; however, both the beam intensity and the energy calibration would be affected, particularly at low angle/high energy. This is un­acceptable for a spectroscopy beamline, where both intensity and energy stability are important. Therefore, a software-based feedback system was developed to simultaneously maximize the intensity and control the beam position. The beam intensity and position before the KB mirrors is measured using two gridded split ion chambers. These were selected over a single-crystal beam position monitor (BPM) to avoid artefacts from electrode patterning, and they serve an additional role in providing a system for recording bulk XAS standards for user experiments. The ion chamber BPM is located at 183 m, and while the position resolution is in the order of micrometres, the large lever arm results in sensitivity to nanoradian-level movements from the primary optics. This scheme is thus robust and allows users to arbitrarily set any energy for scanning within the operating range of the beamline.

## 3. Experimental endstation   

The endstation vessel contains both the KB system and sample stage assemblies. These elements are placed in separated chambers, with a 1 µm-thick silicon nitride membrane acting as a window for the beam to pass through the dividing wall. This allows air, inert gas and vacuum operation on the sample side whilst maintaining a constant protective ultra-high vacuum environment for the focusing optics. Although KB systems can be operated in inert atmospheres, great care must be taken to ensure the atmosphere is clean and dry to guarantee long-term mirror quality. Previous experience at DLS with operation using inert atmospheres has been mixed with some microfocus beamlines showing degradation of the focus and build up on the mirrors within 1 year. The vacuum environment operates at 5 × 10^−8^ mbar, achieved using an ion pump, and mitigates the risk of degradation. It also reduces the risk of any potential room temperature or inert gas temperature related drift.

**Figure 3:**
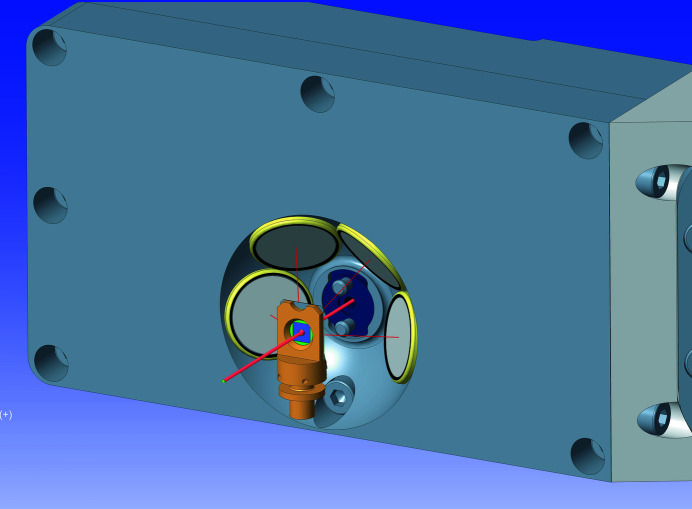
XRF detector and sample holder arrangement. The sample is 17 mm from the centre of each sensor. A pinhole mounting is designed into the central aperture of the detector.

**Figure 4:**
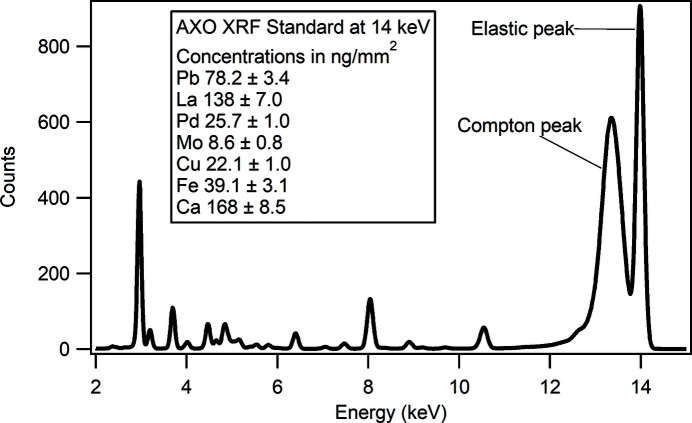
Example XRF spectrum acquired for 1 s acquisition at 14 keV from a known XRF calibration standard. The backscatter geometry results in a strong elastic and Compton peak in this case, but the excellent peak–background performance of the detector reduces any impact on dilute concentration measurements.

The KB vertical and horizontal mirror lengths use a 3:1 ratio to provide a similar numerical aperture (NA) for imaging or out-of-focus measurements such as projection imaging or near-field ptychography. The ratio also better matches the horizontal and vertical coherence length differences resulting from the optical scheme of the beamline. The mechanics of the KB mirrors were designed in-house and use a flexure scheme for angular adjustment based on high-stability monochromator designs developed at DLS. The KB mechanics allow for pitch adjustment on each mirror and roll adjustment on the vertical mirror to ensure that they are perpendicular. Survey and offline optical metrology were used to initially position the mirrors but there are no translational motions as the long lever arm of the beamline allows the beam position to be moved by hundreds of micrometres at the KB with only microradian-scale movements of the primary optics. A four-element XRF detector (RaySpec, UK) in a backscatter geometry is located inside the endstation vessel, with each silicon drift detector (SDD) focused at 17 mm from the sample (Fig. 3 ▸). A hole in the detector allows the beam to pass through and is equipped with a mount for installing pinhole apertures to further clean the beam. The overall arrangement results in a working distance of 6 mm from the front of the detector. The detector pulse processing is performed using Xspress3 (Dennis *et al.*, 2019 ▸; Farrow *et al.*, 1995 ▸) with an overall maximum count rate of 6 mcps. A backscattering geometry is often not considered optimal due to the increased scatter peak signal; however, the design of *in situ* sample environments generally restricts the visibility of the sample at 90° and, in the thin sample regime, the impact of the scatter is acceptable in terms of signal to noise ratio (Sun *et al.*, 2015 ▸). An example spectrum, collected at 14 keV from a calibration standard, is shown in Fig. 4 ▸.

Several additional detectors are installed downstream of the sample on a granite platform for imaging, diffraction and phase contrast/ptychography applications. Two Medipix3 based detectors (Ballabriga *et al.*, 2011 ▸), an Excalibur 3M detector (Marchal *et al.*, 2013 ▸) and a Merlin Quad (Quantum Detectors, UK), are mounted adjacent to each other and used for diffraction measurements or phase contrast imaging. Although the detectors are based on the same technology, the direct beam exposure needed for differential phase contrast (DPC) and ptychography is better managed on a separate smaller detector. For projection imaging, speckle-based imaging and near-field ptychography an effective pixel <55 µm of the Medipix3 is needed, so a scintillator-based detector was built using a sCMOS camera (Andor Neo, Oxford Instruments, Northern Ireland) and a set of standard microscope objectives (Olympus, UK) mounted on a custom-designed rotary stage.

**Figure 5:**
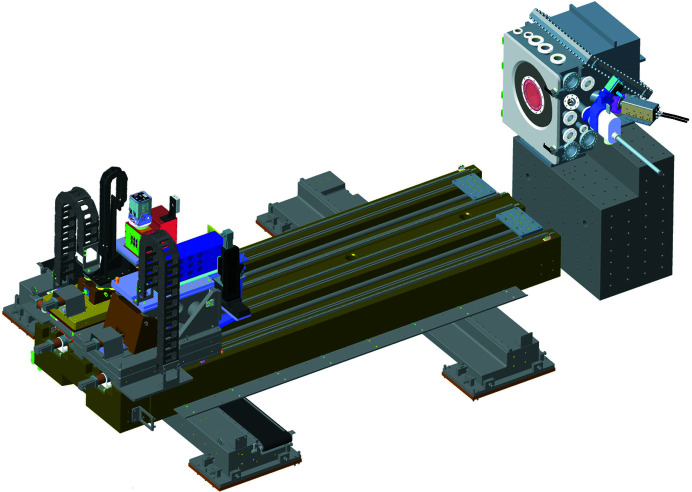
General arrangement of the combined sample/KB vessel and the platform hosting downstream detectors.

Each of these detectors can be placed between 0.2 m and 3.5 m from the sample using independent motorized drives. Motorized lateral motion of the base platform is provided to swap between detector systems in less than 1 min. Each detector also has a compact wedge vertical lift stage, with the scintillator-based camera featuring an additional stage for fine positioning. The general arrangement of the detector platform is shown in Fig. 5 ▸.

The sample is mounted on a post and secured by magnets. Positioning is achieved using a mixture of flexure piezo stages and stepper motor controlled cross-roller stages for fine and coarse control, respectively. This combination of piezo and cross-roller stage is commonly used to achieve nano positioning over a long travel range. The cross-roller based stages typically have resonances in the 50–80 Hz range and the stiffness of the rollers can define the overall stability. For this setup, vibrations at a resonance of 72 Hz are seen with peaks of 4 nm vertically and 10 nm horizontally, respectively. The poorer horizontal performance is in part due to stronger vibrations in this direction from the floor but mainly the stiffness of the overall stack. A calibration chart measured intermittently over a 38 h period showed maximum drifts of 150 nm horizontally and 100 nm vertically, but in practice the authors observed variations in drift which are dependent on the sample and mounting. A compact rotary stage helps to optimize the angular position of flat samples and enables advanced techniques such as multimodal tomography. Locating the sample is achieved by a retractable in-line microscope positioned downstream. This shares the focal plane with the KB system while offering a smaller depth of focus, guaranteeing that if the sample is in focus in this microscope it is also at the X-ray focus. A focal spot size of 50 nm is achieved at 12 keV, measured using ptychography and resolution targets (Fig. 7). The spot size varies, as expected, due to changes in the diffraction limit across the beamline energy range. Flux on the sample, at 12 keV, is approximately 5.4 × 10^9^ photons s^−1^ at the finest focus. Opening the secondary source aperture broadens the horizontal spot size and increases flux. A flux of 5 × 10^10^ photons s^−1^ can be achieved, for example, by increasing to a 250 nm horizontal beam size.

## 4. User operations   

Scanning probe experiments allow for a range of multimodal measurements using different detectors and scan types (*e.g.* ptychography, XRD, XRF, XANES and tomography), so there is a requirement for flexible scanning of detectors, translations, rotations and energy. The broad range of techniques coupled with the wide variety of science applications and heterogeneous, often novice, user base has led DLS to develop an integrated technology stack which can be deployed across the facility. This provides a flexible and easy to use user interface that allows intuitive interaction with the beamline while abstracting hardware details which are not of immediate interest to the user. The system provides experiment control, hardware-based scanning and triggering, task scheduling, and automated processing for agile feedback during the experiment (Basham *et al.*, 2018 ▸). It is also fully scriptable so that complex experiments and new techniques can be implemented quickly.

Verification of the focal spot size and re-focusing of the KB, if required, are performed before each user experimental session using ptychographic reconstruction of the sample or a reference target.

In a typical experiment, once the sample is mounted on the stage, the first operation is to position the sample at the X-ray focus using the in-line microscope and interactive controls for the sample stages to find an area of interest with. A marker in the image indicates the approximate position of the X-ray beam within ∼1 µm. The scan is defined by drawing a region of interest on the captured frame from the camera and selecting either the number of points required or the step size in each direction, as well as whether scanning should be continuous or step by step. After choosing the detector combination and the dwell time the scan can be submitted to the queue which manages the hardware required for data acquisition.

Before launching the scan, the microscope is retracted, and the appropriate detector is moved into place. The complex motion sequence is carried out by a script in order to avoid potential collisions. Prior to submission, the user can optionally define one or more ‘processing chains’ which run continuously and process detector data as they arrive, providing direct feedback on experiment progress and dynamic data processing and visualization. Typical examples are peak integration to select elements of interest from the XRF signal, or frame processing for differential phase contrast. Because all data, including the microscope image, share the same coordinate space, data from different sample regions can be overlaid and displayed simultaneously. An intuitive point-and-click interface allows interactive inspection of both raw and processed data of any point in a scan. The online data processing and integrated visualization are key elements of the experiment workflow, since they enable progressive refinement of the scan area. Thus, typically a coarse scan with 500–750 nm step sizes is launched over a relatively large area, providing a broad overview of the sample at a resolution comparable to the optical microscope. Based on the processed results, the step size can be progressively refined over smaller areas, providing a ‘zoom’ effect with increasing spatial resolution.

**Figure 6:**
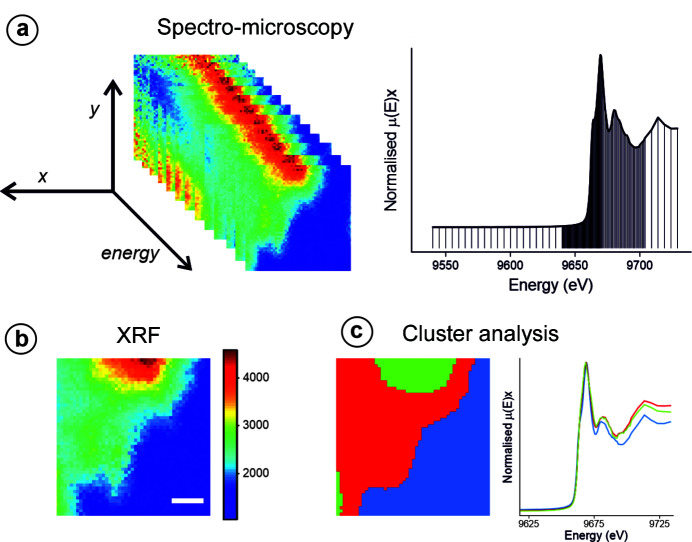
(*a*) Spectro-microscopy methodology for speciation analysis, based on acquiring multi-energy 2D fluorescence maps along the absorption edge of the target element. In this experiment 135 energies across the Zn *K*-edge were scanned. (*b*) XRF map (Zn *K*α emission) collected above the Zn *K*-absorption edge (*E* = 9,669 eV). Scale bar length: 750 nm. The numbering on the colour scale bar represents fluorescence intensity (arbitrary units). (*c*) Cluster analysis representation revealing statistically similar regions according to their XANES spectra. Image reprinted (adapted) with permission from Gomez-Gonzalez *et al.* (2019 ▸). Copyright 2019 American Chemical Society.

**Figure 7:**
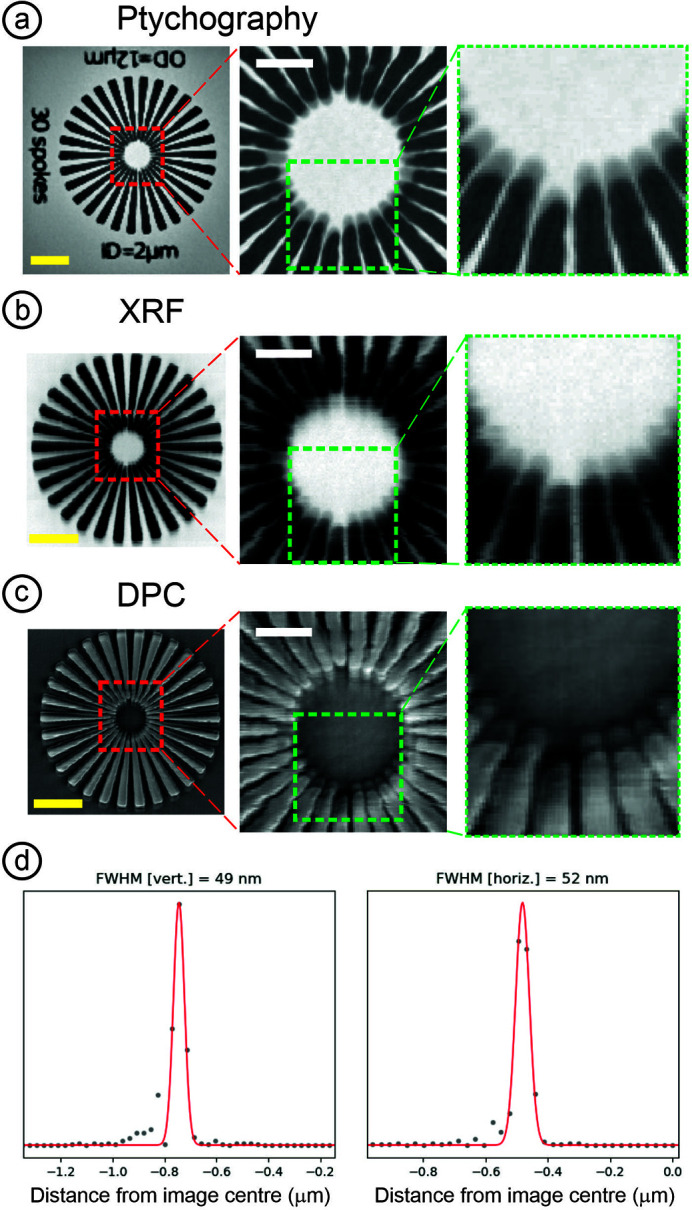
Multimodal imaging of a tungsten Siemens Star with 70 nm minimum feature size using (*a*) ptychography, (*b*) XRF and (*c*) DPC. (*d*) A typical beam profile extracted from ptychography reconstructions at 12 keV. The ptychography scan was acquired out of focus, covering a 16 µm × 16 µm area. The zoomed-in area shows the detail of the high-resolution features in the reconstruction. XRF (*L*α emission) and DPC maps of the Siemens Star were acquired simultaneously at focus with 50 ms dwell time and 40 nm scan steps per pixel. Scale bars in all pictures are 1 µm. The pixel size in the ptychographic reconstructions is 27 nm.

Early beamline publications (McCulloch *et al.*, 2019 ▸; Gomez-Gonzalez *et al.*, 2019 ▸; Morrell *et al.*, 2019 ▸; Walker *et al.*, 2018 ▸) highlight some of the XRF, nano-XANES and nano-XRD capabilities. Fig. 6 ▸ shows nano-XANES maps acquired on ZnO nanorods which were incubated *in situ* in a simulated sludge solution (Gomez-Gonzalez *et al.*, 2019 ▸). In this experiment, a spectro-microscopy methodology for speciation analysis was followed, based on multiple 2D XRF maps acquired around and across the Zn *K*-edge. The XANES data were aligned and processed in a similar fashion to STXM, using PCA and cluster analysis to extract variations in the recorded sample spectra [Fig. 6 ▸(*c*)] (Lerotic *et al.*, 2004 ▸, 2014 ▸). As a simple example of the ptychography and general imaging capabilities, Fig. 7 ▸ shows XRF, DPC and ptychography images of a 1 µm × 1 µm area from a tungsten test object with 50 nm feature spacing.

## 5. Conclusions   

We have designed, constructed and commissioned a high-resolution versatile scanning microscope suitable for multimodal-imaging in the hard X-ray regime with particular emphasis on energy scanning for XAS-based measurements. The Nanoprobe supports X-ray fluorescence, transmission diffraction, differential phase contrast, ptychography and tomography imaging techniques with a nominal beam size of 50 nm × 50 nm allowing I14 to tackle problems in a diverse range of scientific areas such as life, geological, environmental and materials sciences. The instrument is operational and open to the general user community.

## Figures and Tables

**Table 1:** Summary of the key components at I14

Component	Distance (m)	Supplier	Parameters and comments
Primary slits	21	IDT	–
M1	26	IRELEC	1 m-long, Si, Rh, Pt stripes. Cooling via Ga eutetic
M2	27.5	IRELEC	1 m-long, Si, Rh, Pt stripes. Cooling via Ga eutetic
DCM	35	IDT horizontally deflecting monochromator	Si(111) 60 mm and 200 mm crystals
Diamond single-crystal BPM	53	Cividec	3 mm 50 µm diamond with Ti anodes
Horizontal secondary source	55	IDT	Flexure parallel opening mechanism with cylindrical blades
Feedback BPM	183	ADC	Two 50 mm gridded split ion chambers
Beam defining slits	183.5	JJ slits	Scatterless GaAs blades
Nano KB	184	JTEC mirrors/in-house mechanics	180 mm (V) and 60 mm (H) long with Pt/Rh bilayer
XRF detector	–	Rayspec	Four 50 mm^2^ Ketek SDDs with cube preamplifiers.
Sample stage	–	Piezosystem Jena/Physike Instrumente/Attocube	–
Detector table	185–188	In house	–
